# Characterization of Hydrophilic Polymers as a Syringe Extrusion 3D Printing Material for Orodispersible Film

**DOI:** 10.3390/polym13203454

**Published:** 2021-10-09

**Authors:** Pattaraporn Panraksa, Sheng Qi, Suruk Udomsom, Pratchaya Tipduangta, Pornchai Rachtanapun, Kittisak Jantanasakulwong, Pensak Jantrawut

**Affiliations:** 1 Department of Pharmaceutical Sciences, Faculty of Pharmacy, Chiang Mai University, Chiang Mai 50200, Thailand; pattaraporn.prs@gmail.com (P.P.); ptipduangta@gmail.com (P.T.); 2 School of Pharmacy, University of East Anglia, Norwich NR4 7TJ, UK; sheng.qi@uea.ac.uk; 3 Biomedical Engineering Institute, Chiang Mai University, Chiang Mai 50200, Thailand; suruk_u@cmu.ac.th; 4 Division of Packaging Technology, Faculty of Agro-Industry, School of Agro-Industry, Chiang Mai University, Chiang Mai 50100, Thailand; pornchai.r@cmu.ac.th (P.R.); jantanasakulwong.k@gmail.com (K.J.); 5 Cluster of Agro Bio-Circular-Green Industry (Agro BCG), Chiang Mai University, Chiang Mai 50100, Thailand

**Keywords:** syringe extrusion 3D printing, orodispersible film, hydrophilic polymer, disintegration

## Abstract

The application of hydrophilic polymers in designing and three-dimensional (3D) printing of pharmaceutical products in various dosage forms has recently been paid much attention. Use of hydrophilic polymers and syringe extrusion 3D printing technology in the fabrication of orodispersible films (ODFs) might hold great potential in rapid drug delivery, personalized medicine, and manufacturing time savings. In this study, the feasibility of 3D-printed ODFs fabrication through a syringe extrusion 3D printing technique and using five different hydrophilic polymers (e.g., hydroxypropyl methylcellulose E15, hydroxypropyl methylcellulose E50, high methoxyl pectin, sodium carboxymethylcellulose, and hydroxyethylcellulose) as film-forming polymers and printing materials has been investigated. Rheology properties and printability of printing gels and physicochemical and mechanical properties of 3D-printed ODFs were evaluated. Amongst the investigated hydrophilic polymers, sodium carboxymethylcellulose at a concentration of 5% *w*/*v* (SCMC-5) showed promising results with a good printing resolution and accurate dimensions of the 3D-printed ODFs. In addition, SCMC-5 3D-printed ODFs exhibited the fastest disintegration time within 3 s due to high wettability, roughness and porosity on the surface. However, the results of the mechanical properties study showed that SCMC-5 3D printed ODFs were rigid and brittle, thus requiring special packaging to prevent them from any damage before practical use.

## 1. Introduction

3D printing (3DP) technology has recently been intensively researched for its potential as a designing and manufacturing method in the pharmaceutical field to fabricate individualized pharmaceutical products that are not available through traditional manufacture. 3DP offers the ability to tailor the design (shape and size) and drug dosing on-demand further to suit the individual clinical needs of each patient [1]. Various 3DP techniques have been reported regarding the fabrication of pharmaceutical dosage forms, including binder jetting [2], stereolithography [3], selective laser sintering (SLS) [4], inkjet printing [5], and extrusion-based printing [6,7]. Extrusion-based printing works based on using the pneumatic air pressure or mechanical screw systems to extrude the printing materials through a needle or nozzle layer-by-layer [8]. In the pharmaceutical field, syringe-based semi-solid extrusion (SSE) and fused deposition modeling (FDM) are the two main extrusion-based printing techniques used [9]. Syringe extrusion 3D printing has been applied across a range of research fields, with particular advantages over other printing techniques in terms of cost-effectiveness, the wide availability of printing materials, and room temperature printing capability for thermo-labile drugs [10]. A range of dosage forms have been designed and fabricated using syringe extrusion 3D printing, such as immediate-release tablets, local drug delivery patches, contraceptive vaginal rings, and oromucosal films (buccal films and orodispersible films) [11,12,13,14,15,16,17].

Orodispersible films (ODF) are one of the promising personalized dosage forms of choice for pediatric patients and geriatric patients suffering from dysphagia, severe psychological or neurological disorders [18]. This dosage form is often made from hydrophilic polymers and can be administered orally and then disintegrate rapidly in the buccal cavity without requiring water, leading to rapid dissolution and fast onset of action [19]. To date, various natural and synthetic hydrophilic polymers, e.g., hydroxypropyl methylcellulose (HPMC), polyvinyl alcohol (PVA), polyethylene oxide (PEO), maltodextrin, and pregelatinized starch, have been investigated to fabricate ODFs for an individualized therapy by different extrusion-based 3D printing methods. For instance, in a study by Jamróz et al. [20], PVA was successfully used for the preparation of FDM filaments loaded with aripiprazole and the FDM printed ODFs exhibited good mechanical properties and fast disintegration within 1 min. In 2018, the FDM 3D printer was employed to fabricate the single-layered FDFs (SLFDFs) and multilayered FDFs (MLFDFs) films made of PEO with ibuprofen or paracetamol and PVA with paracetamol in plain or mesh designs. Mesh SLFDFs and mesh MLFDFs had rapid disintegration times of 42 and 48 s, respectively [21]. Furthermore, in 2021, the FDM 3D printer was investigated in another study to produce the individualized cannabidiol ODFs. This study proposed a method for promoting automation in the manufacturing of personalized medicines [22]. In another study by Elbl et al. [23], the multilayered ODF maltodextrin films of benzydamine hydrochloride were successfully fabricated using an in-house modified syringe extrusion 3D printer. In this study, the addition of different viscosity grades of hydroxyethylcellulose was used to adjust the viscosity of the ink in order to achieve successful printing with in-process drying. Furthermore, in a study conducted by Sjöholm et al. [24], personalized warfarin ODFs were successfully fabricated using semisolid extrusion (EXT) 3D printing, and sufficient therapeutic doses of warfarin were achieved.

For syringe extrusion 3D printing, there is a range of important factors that need to be considered for the selection of polymers, such as rheological characteristics, extrudability through the nozzle, reproducible and consistent flow of the printing formulations, and drying conditions [25]. Even though there are studies that show various hydrophilic polymers can be potentially applied as printing materials, there are still limited studies that attempt to explore and evaluate the possibility of using the hydrophilic polymers as the printing materials for syringe extrusion 3D printing. Moreover, there are few studies available on the fabrication of ODF using syringe extrusion 3D printing. As a result, the purpose of this research was to investigate and identify the best printing material for syringe extrusion 3D printing, which can be used not only for ODF fabrication but also for other pharmaceutical applications such as oral drug delivery and immediate release drug delivery systems. In this study, the physicochemical and mechanical properties of the syringe extrusion 3D printed placebo ODFs were investigated in detail. This allowed us to evaluate the potentials of hydrophilic polymers including hydroxypropyl methylcellulose E15, hydroxypropyl methylcellulose E50, high methoxyl pectin, sodium carboxymethylcellulose, and hydroxyethylcellulose as matrix printing material for ODF formulations.

## 2. Materials and Methods

### 2.1. Materials

Hydroxypropyl methylcellulose E15 (HPMC E15, AnyCoat^®^-C AN15, substitution type 2910, viscosity 15 mPa·s) was purchased from Lotte Fine Chemical Co., Ltd. (Seoul, Korea). Hydroxypropyl methylcellulose E50 (HPMC E50, substitution type 2910, viscosity 50 mPa·s) was purchased from Zhongbao Chemicals Co., Ltd. (Hangzhou, China). High methoxyl pectin (HMP, degree of esterification = 60%) was purchased from Du Pont^®^ (Wilmington, DE, USA). Sodium Carboxymethylcellulose (SCMC) and hydroxyethylcellulose (HEC) were purchased from Kima Chemical Co., Ltd. (Shandong, China). Distilled water was used as the solvent for preparing the printing gels. All of the other reagents were analytical grade.

### 2.2. Fabrication of 3D-Printed Orodispersible Films

The 3D-printed ODFs were prepared with three different concentrations of each polymer (HPMC E15, HPMC E50, HMP, SCMC, and HEC) as shown in Table 1. The concentrations of each polymer were selected based on the flowability through the nozzle of printing formulations. At minimum effective concentration, the printing gels should be able to create the continuous flow of printing gels and should not flow as liquids due to too low of viscosity. The printing gels were prepared by dispersing each polymer in deionized water at 60 ± 0.5 °C and stirred for 3 h. Then, the polymer solutions were cooled down to room temperature, after which methylene blue at a concentration of about 1.0% *w*/*v* of total formulation was added in order to make the printed-ODFs more visible and easier to analyze their printing accuracy by the image analysis software. All polymer gels were left to stand until the air bubbles disappeared and subsequently transferred into a 10 mL disposable syringe for printing.

In this study, the 3D printing process was performed at room temperature by the custom-built in-house syringe extrusion 3D printer [26], which was developed and constructed by the Biomedical Engineering Institute (BMEI) of Chiang Mai University. This 3D printer was designed based on the principle of the extrusion-based 3D printing technique. The printer is a Core XY-based 3D printer and consisted of a movable syringe system, building platform, stepper motor, screw device, and computer and user-interface control system. The syringe system, which is comprised of a plunger of a 10 mL syringe (14.5 mm in internal diameter) and a removable extrusion nozzle, is movable in the X and Y directions by the stepper motor via a direct lead screw drive. In addition, there is a separate building plate moving on the Z-axis to preventing the vibration on printed objects. Before 3D printing, an object was designed using an open-source program and was divided into numerous two-dimensional (2D) layers with a defined thickness, infill and speed of printing. These 2D layers can be piled up by selectively adding the desired materials in a highly reproductive layer-by-layer manner under the instruction of computer-aided design (CAD) models.

**Figure 1:**
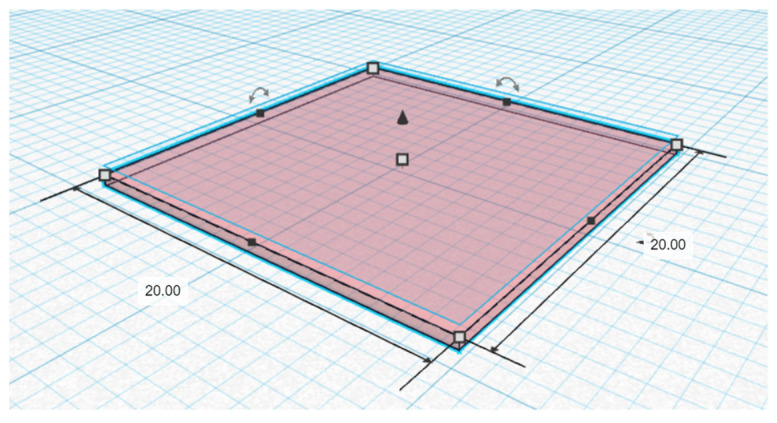
CAD model of the 3D-printed ODF.

The model of the 3D-printed ODF with the dimension of 20.0 mm width × 20.0 mm length × 0.50 mm height (Figure 1) was designed using Tinkercad^®^ software (2021, Autodesk Inc., San Rafael, CA, USA). The 3D or CAD file was exported to Repetier-Host software version 2.1.6 (Hot-World GmbH & Co. KG., Willich, Germany) as a 3D printer readable stereolithography (.stl) file format. The .stl file was converted to a 3D printable code (G-code) by the open-source Slic3r software version 1.3.1 (GNU Affero general public license, version 3), and the 3D model was subsequently sliced into two layers of 2D horizontal cross-sections with a defined thickness of 0.25 mm. Afterward, the 3D-printed ODFs were fabricated with the syringe extrusion 3D printer by extruding the printing gels through a 21-gauge size extruder nozzle (0.51 mm in diameter). The printing parameters were set as follows: layer height = 0.25 mm, perimeters = 2, fill angle = 45°, speed of printing = 10 mm/s, traveling speed of nozzle = 120 mm/s, and the infill was defined as rectilinear with 100% ratio. The whole printing process was conducted within 2 min 48 s under the constant temperature of 25 °C. After printing, the 3D-printed ODFs were left for drying at room temperature for 6 h.

### 2.3. Rheological Characterization

The rheological behaviors and viscosities of all printing gels were investigated by means of a parallel-plates rheometer with a P25 DIN plate (Brookfield Rheometer R/S, Brookfield Engineering Laboratories, Middleboro, MA, USA). The gap between plate and platform was set at 1 mm. All the experiments were carried out at 25 °C in triplicate. The shear stress and viscosity were obtained using the CSR (Controlled Shear Rate) mode with the shear rate ranging from 0 to 100 s^-1^. Then, the experimental data were fitted with the power-law model, and the flow behavior index (n) and consistency coefficient (K) values were determined by Equation (1):

(1) $$\tau =K{\dot{\gamma }}^{n}$$

where τ is the shear stress (Pa), $\dot{\gamma }$ is the shear rate ($s^{-1}$), n is the flow behavior index, and K is a consistency coefficient ($Pa\cdot s^{n}$).

### 2.4. Dimensional Accuracy Analysis

In this study, the dimensional accuracy analysis was performed immediately after drying and post-drying in order to determine the printability, dimensional stability, and accuracy of the 3D-printed ODFs. The dimensional accuracy analysis consisted of two parts: the diameter test and the shape fidelity test. For the diameter test, the printed filaments were fabricated by setting the infill parameter as 0% infill, the perimeter as 1, and the other printing parameters as described in Section 2.2. 

The digital images of the printed filaments and ODFs were taken by a digital camera (Canon EOS 750D with an 18–55 mm lens, Canon, Inc., Tokyo, Japan) and then analyzed by an image processing program (Image J version 1.53, National Institutes of Health, Bethesda, MD, USA). The shape fidelity factor (SFF), which is the ratio between the 3D-printed surface area and CAD model area, was calculated [27].

### 2.5. Morphological Characterizations

The morphology of the 3D-printed ODFs was investigated by visual inspection and scanning electron microscopy (SEM) with a JEOL scanning electron microscope (JSM-5410LV, JEOL Ltd., Peabody, MA, USA) at 10 kV under low vacuum mode. The 3D-printed ODFs characterizations were performed without any coating solution at magnifications of ×500. The cross-sectional and thickness of films were evaluated. The surface morphology of the 3D-printed ODFs were examined by using an atomic force microscopy (AFM) instrument (XE70 model, Park Systems Corporate, Suwon, Korea) in contact mode with NCS36 cantilevers, with tip apex radius of curvature under 10 nm, the scan rate of 1 Hz and scan area of 5 × 5 μm^2^. AFM parameters such as average surface roughness (Sa), root mean square (RMS) surface roughness (Sq), maximum peak height (Sp), and maximum pit depth (Sv) were determined.

### 2.6. Weight and Thickness

For each formulation, five 3D-printed ODFs were selected randomly. The 3D-printed ODFs were weighed individually using an analytical balance (LAB 214i, Adam Equipment Co., Ltd., Jing An, Shanghai, China). The thickness was assessed at three different points of each film using an outside micrometer (3203-25A, Insize Co, Ltd., Suzhou New District, Jiangsu, China). The average weight and thickness of each formulation and their standard deviation (SD) were calculated.

### 2.7. Mechanical Strength Test of 3D-Printed ODFs

The mechanical properties of the 3D-printed ODFs were determined using a texture analyzer TX.TA plus (Stable Micro Systems, Surrey, UK) equipped with a 5 kg load cell (0.001 N of sensitivity) and a 2 mm stainless steel cylinder probe (P/2 plane ﬂat-faced surface probe), similar to a previous study [26]. The 3D-printed ODF was fixed on a heavy-duty platform (HDP/90) with a cylindrical hole of a diameter of 9.0 mm (the surface area of the cylindrical hole was 63.56 mm^2^). The probe was moved down until it penetrated the film to a distance of 10.0 mm. The texture analyzer was set at a pre-test speed of 1.0 mm/s, a test speed of 1.0 mm/s, and a post-test speed of 10.0 mm/s to measure force in the compression mode. All tests were performed in triplicate for each formulation. The maximum force (N), distance (mm), and slope of the linear region of the force-time curve (N/s) were recorded. Thereafter, the mechanical parameters (tensile strength, percent elongation, and Young’s modulus) were calculated [28,29]. The obtained tensile strength and percent elongation were normalized by the average cross-sectional area of 3D-printed ODFs.

### 2.8. In Vitro Disintegration Time Study

The in vitro disintegration time study for the 3D-printed ODFs was carried out using a modified disintegration test system described by Preis et al. [30]. The 3D-printed ODF was firmly clamped on the top side with the sample holder and attached with the 3 g magnetic clip on the bottom side of the film. The magnetic clip, which weighs about 3 g (0.03 N), was used to represent the approximate minimal force applied by the human tongue. Then, the attached film was placed half immersed in 65 mL of simulated salivary fluid (SSF) pH 6.8, which was prepared according to Marques et al. [31], at 37 ± 0.5 °C. The disintegration time of the 3D-printed ODF was recorded visually when the magnetic clip dropping down. All studies were carried out in pentaplicate for each formulation. The obtained data were normalized by the average 3D-printed ODFs thickness. For the normalization, the obtained disintegration time of each film was divided by the ratio between its thickness and the minimum values of average thickness of all formulations.

### 2.9. Water Contact Angle Measurement

The water contact angles (θ) of the 3D-printed ODFs were measured using a drop shape analyzer (DSA30, KRÜSS GmbH, Hamburg, Germany). The water droplet with the volume of 20 ± 0.5 µL was dropped onto the planar surface of the 3D-printed ODFs and the images at different time points (0 s and after 30 s) were taken and evaluated. All measurements were done in triplicate for each formulation at room temperature (25 ± 2 °C).

### 2.10. Statistical Analysis

All data were expressed as mean ± standard deviations (SD). The one-way ANOVA test and independent *t*-test were carried out using SPSS^®^ statistics software version 17.0 (IBM Corporation, Armonk, NY, USA) to analyze the statistical significance of the results. The *p* level less than 0.05 were considered statistically different.

## 3. Results

### 3.1. Rheological Behaviors and Dimensional Accuracy Analysis

**Figure 2:**
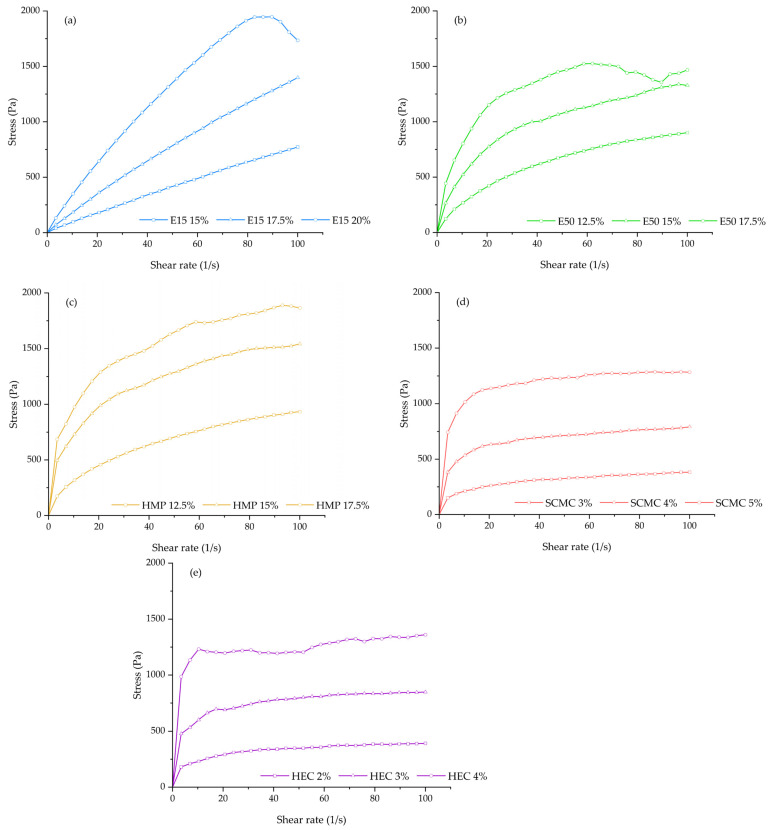
Rheogram of (**a**) HPMC E15 printing gels at concentrations of 15, 17.5, 20% *w*/*v*; (**b**) HPMC E50 printing gels at concentrations of 12.5, 15, 17.5% *w*/*v*; (**c**) HMP printing gels at concentrations of 12.5, 15, and 17.5% *w*/*v*; (**d**) SCMC printing gels at concentrations of 3, 4, and 5% *w*/*v*; and (**e**) HEC printing gels at concentrations of 2, 3, 4% *w*/*v*.

The flowability and viscosity characteristics of the printing gels are one of the factors that need to be considered for the polymer selection in order to enable the continuous 3D printing process. The results of rheological studies showed that all printing formulations behaved as the non-Newtonian fluid and exhibited the shear-thinning (pseudoplastic) flow that is a desirable rheological property for extrusion-based 3D printing [32,33]. As observed from the rheogram (Figure 2), the relationship between the shear-stress and shear-rate of all printing gels was non-linear and had no direct proportionality. The shear stress was found to decrease when the shear rate increased from 0 to 100 s^-1^, thus indicating the presence of non-Newtonian behavior for all printing gels; whilst the apparent viscosity values of all printing formulations were found to significantly decrease with increasing of shear-rate, with the flow behavior index (n) of less than 1 (0.07–0.90), thereby indicating the pseudoplastic (shear-thinning) nature of all investigated polymers and allowing easy extrusion through the nozzle. Furthermore, this study also showed that the high consistency coefficient (K) was observed when the concentration of printing polymer increased, indicating that the printing gels became more viscous and more pseudoplasticity at higher concentrations. Thus, requiring more energy and higher force to extrude the printing gels through the narrow nozzle. Recently, it has been reported that the printing gels with higher viscosity seemed to be strongly advantageous in terms of resolution and quality improvement as well as the accuracy of the 3D-printed ODFs structure [34].

In this study, the results also showed that the printability of our syringe extrusion 3D printer was strongly affected by the rheological characteristics and viscosity of each printing formulation. As can be seen in Table 2, the diameters of printed filaments (immediately after printing) were found to be decreased with the increase of polymer concentration and viscosity. The diameters of printed filaments (Figure S1) were mostly close to the actual diameter of extrusion nozzle (0.51 mm) at the concentrations of 20% *w*/*v* for HPMC E15, 15% *w*/*v* for HPMC E50, 15% *w*/*v* for HMP, 5% *w*/*v* for SCMC, and 4% *w*/*v* for HEC (0.57 ± 0.07, 0.53 ± 0.03, 0.59 ± 0.01, 0.56 ± 0.02, and 0.56 ± 0.05 mm respectively), proving that the good printing resolution and the accuracy of printing process were achieved. However, the printing gels with too high concentration were not favorable for syringe extrusion 3D printing due to the nozzle blockages and intermittent extrusion issues. According to the dimensional accuracy analysis experiments, it was observed that the printing of E50-17.5 was failed due to the extrusion issues at this high concentration. The E50-17.5 printing gels could not extrude through the nozzle (size 0.51 mm in diameter). Furthermore, it was also observed that the diameters of HMP-17.5 printed filaments were found to be narrow than the actual nozzle diameter. That means that upon the further addition of HMP concentration from 15 to 17.5% *w*/*v*, the right amount of the printed filament could not be extruded from the nozzle due to too high viscosity. 

For the dimensional stability of the printing gels after printing, the shape fidelity factor (SFF) of these 5 formulations (E15-20, E50-15, HMP-15, SCMC-5, and HEC-4) was close to one, confirming that the structure of 3D-printed ODFs matched with the original CAD model. However, we observed that after post-drying the HEC-4 3D-printed ODFs could not be peeled off from the building plate without ripping whilst the other 4 formulations could. When the dimensional accuracy of post-drying 3D-printed ODFs was measured, we found that the shape fidelity of E15-20, E50-15, HMP-15, and SCMC-5 3D-printed ODFs was slightly decreased when compared to those after printing but remained close to one, indicating that the 3D-printed films could stack up in two layers and the solid polymer still maintained the printed structure without sagging or structural deformation. The shape fidelity was determined to be 0.99 ± 0.01, 0.99 ± 0.03, 1.00 ± 0.01, and 0.98 ± 0.02 for E15-20, E50-15, HMP-15, and SCMC-5 3D-printed ODFs, respectively. The slight reduction in the peripheral dimensions of the dried 3D-printed ODFs could be attributed to the solvent evaporation process. As the solvent (ethanol and water) evaporated during drying process, the polymer chains were drawn closer together and packed densely, forming the solid-like and stable shape of the films. Consequently, the results of this study indicated that, for the polymer tested in this study, the suitable viscosity range to achieve good dimensional accuracy and resolution of the 3D-printed ODFs is 26–44 Pa·s. Thus, the E15-20, E50-15, HMP-15, and SCMC-5 formulations were subsequently selected for further evaluation for their physicochemical and mechanical properties of the 3D-printed ODFs.

### 3.2. Morphology of 3D-Printed ODFs

**Figure 3:**
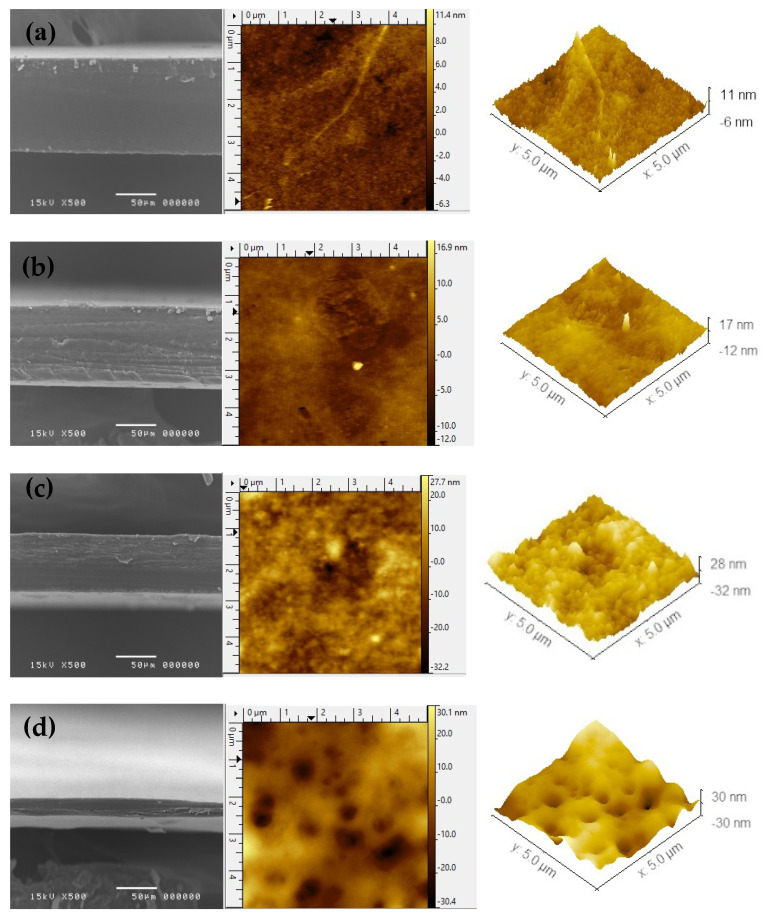
Cross-sectional SEM (left), 2D height AFM topography (middle) and the corresponding 3D AFM topography (right) images of (**a**) E15-20; (**b**) E50-15; (**c**) HMP-15; (**d**) SCMC-5 printed ODFs.

Imaging methods of examining morphologies, such as atomic force microscopy (AFM) or scanning electron microscopy (SEM), are widely employed in the characterization of thin-film materials [35,36]. In this study, cross-sectional SEM images and two-, three-dimensional (2D, 3D) surface topography of AFM can provide quantitative information about the pores present in the polymer matrix and on the sample surface, respectively. The cross-sectional SEM images of 3D-printed films along with the surface morphologies examined by AFM are shown in Figure 3. From the cross-sectional SEM images, all 3D-printed ODFs are dense without any pores within polymer matrix. The average film thickness obtained from the SEM observation is ~90 µm for E15-20, ~70 µm for E50-15, ~50 µm for HMP-15, and ~20 µm for SCMC-5 3D-printed ODFs. The thickness from micrometer measurement was slightly different in comparison to that obtained using SEM because of the variation between different instruments. However, the thickness from both instruments showed the same trend.

The AFM technique provides information about the microscopic topology of 3D-printed ODFs. As seen in Figure 3, the AFM topography was also used to find out the average surface roughness (Sa), root mean square (RMS) surface roughness (Sq), maximum peak height (Sp), and maximum pit depth (Sv). The results of all these parameters are given in Table 3. From the AFM images and parameters of 3D-printed ODFs, the Sa and Sq of SCMC-5 3D-printed ODFs were found to be 8.83 and 6.96 nm, respectively, which suggested better average surface roughness in comparison to other 3D-printed ODFs. Moreover, SCMC-5 3D-printed ODFs showed higher Sp (30.09 nm) and Sv (32.28 nm) than the others, while E15-20 3D-printed ODFs showed lower Sa (1.51 nm), Sq (1.13 nm), Sp (11.50 nm), Sv (6.33 nm) than the others 3D-printed ODFs. From the AFM results, it is indicated that SCMC-5 3D-printed ODFs represented more pores and rough surface on the film surface than the others. The formation of pores in polymer thin films used in drug delivery systems is important as they have been reported as a higher surface roughness led to faster drug release [37].

### 3.3. Thickness and Weight Variation of 3D-Printed ODFs

The thickness and weight variation of selected 3D-printed ODFs were carried out in order to ensure the consistency of printing process and printed objects. Each film printed with the custom-built in-house syringe extrusion 3D printer showed small standard deviations regarding thickness and weight. The average thickness of selected 3D-printed ODFs varied in the range of 18-89 µm, while the average weight varied in the range of 10-41 mg. As can be observed in Table 4, the average weight of 3D-printed ODFs was increased with the polymer concentration used. No significant difference (*p* > 0.05) was observed between the weight of E50-15 and HMP-15 3D-printed ODFs at 15% *w*/*v* of polymer, whilst a statistically significant difference (*p* < 0.05) was observed when the thickness of E50-15 and HMP-15 3D-printed ODFs were compared. Thus, this study indicated that the thickness of each 3D-printed ODFs is considered to be varied not only by the polymer concentration but also by the type of polymer. Furthermore, we also observed the significant difference in thickness between the dried 3D-printed ODFs and CAD model (0.50 mm). This could be due to mass loss of the solvent (water and ethanol). The solvent evaporation process appeared to be capable of uniformly reducing the film thickness across the film. However, the drastic reduction in post-drying film thickness had no effect on the peripheral dimensions or shape fidelity of the 3D-printed ODFs. After drying, the shape fidelity of the E15-20, E50-15, HMP-15, and SCMC-5 3D printed ODFs sill remained close to one, as mentioned in Section 3.1. These thickness and weight variation results showed that our custom-built in-house syringe extrusion 3D printer can be a potential candidate to be used in a pharmacy or hospital setting to extemporaneously produce ODF dosage form with accurate film weight.

### 3.4. Mechanical Characteristics of 3D-Printed ODFs

The mechanical properties are the important factors to be considered in determining the post-manufacturing handling of the 3D-printed ODFs. Previous studies showed that mechanical properties of ODF films were dependent on polymer grade and also could be influenced by formulation characteristics, e.g., concentration of polymer used or possible excipients blends [38]. The obtained tensile strength, elongation at break, which were normalized by the cross-sectional area of each 3D-printed ODF and their Young’s modulus, are presented in Table 5. Although the Young’s modulus is affected by the cross-sectional area of the sample, the gauge length of the test specimen was maintained constant in order to minimize the impact of having samples with different cross-sectional areas. The result demonstrated that the normalized tensile strength was a statistically insignificant difference between the tested 3D-printed ODFs. However, the 3D-printed ODFs exhibited a variation of elongation and elastic modulus which is probably due to the different nature of the polymer and its concentration. In this study, all 3D-printed ODFs exhibited a slightly different very low normalized elongation range from 2.10 to 3.76%. The Young’s modulus of selected 3D-printed ODFs was in the range of 245 to 333 MPa. HMP-15 and SCMC-5 3D-printed ODFs correspond to the most rigid films (higher Young’s modulus), whereas the E15-20 printed film corresponds to less stiff film (lower Young’s modulus). It is worth mentioning that the HMP-15 and SCMC-5 printed films are hard and brittle (high Young’s modulus and low elongation), thus lowering their handling safety, which may require a special packaging. Considering the structures of pectin, SCMC and HPMC, HPMC presents less available hydroxy groups to establish intra- and inter-polymer chains hydrogen bonds, which could justify the lower rigidity of the HPMC-based films.

### 3.5. In Vitro Disintegration Time

For the direct comparisons of in vitro disintegration times, the obtained disintegration times were normalized by thickness and are presented in Table 6. The results show that the disintegration time for selected 3D-printed ODFs ranged from 2 to 10 s. SCMC-5 and HMP-15 3D-printed ODFs exhibited shorter disintegration time (2.08 and 2.63 s, respectively) than E15-20 (10.62 s) and E50-15 (7.11 s) 3D-printed ODFS, respectively. These results are in good agreement with the AFM data in Section 3.2, in which SCMC-5 3D-printed ODFs showed the highest roughness and porousness on the 3D-printed ODFs surface when compared to others. More porousness and surface roughness may allow water molecules to penetrate, resulting in faster drug release [39] and polymeric matrix disintegration. Furthermore, the increase of the polymer concentration was associated with the increase in the disintegration time of the film. Even though hydrophilic polymers are used in this study, due to relatively high polymer concentration, the viscosity of polymeric matrices increases which results in the formation of a high viscosity gel layer on wetting. The high viscosity gel layer tends to decrease the mobility of polymeric molecules in swollen matrices resulting in an increase in the disintegration time of the film [40,41]. Not only polymer concentration, but its molecular weight, degree of hydrolysis, and hydrogen bonding tendency toward an aqueous solution also influence the disintegration of film [42]. At the same 15% *w*/*v* polymer concentration, the film prepared using HMP (molecular weight = 194.14 g/mol) at 15% *w*/*v* showed a faster disintegration time than the film prepared using HPMC E50 (molecular weight ~90,000 g/mol). This may be due to the lower molecular weight of HMP as compared to HPMC E50.

### 3.6. Water Contact Angle

Water contact angle measurements of the 3D-printed ODFs were carried out in order to investigate the surface wettability of films by an aqueous medium. The water contact angle (θ) is defined as the degree to which the water droplet interacts with the surface of 3D-printed ODFs. Typically, the films with hydrophilic and high surface wetting characteristics display the water contact angles of less than 90°. Conversely, the water contact angles greater than 90° indicates the hydrophobic and low surface wetting characteristic of films [43]. The water contact angles of the 3D-printed ODFs of this study are summarized in Table 6. It was observed that all 3D-printed ODFs (E15-20, E50-15, HMP-15, and SCMC-5) exhibited hydrophilic characteristics with contact angles of less than 90°. Initially, SCMC-5 3D-printed ODFs showed the lowest water contact angle of about 51.5°, whereas E15-20 showed the highest water contact angle of about 62.4°. The decreasing water contact angles of all 3D-printed ODFs were also observed during the measurements. This may be due to the water droplet spreading on the film surface, film disintegration and water penetration into the film [44]. However, there was only a significant decrease in the water contact angle of SCMC-5 3D-printed ODFs after 30 s (*p* < 0.05) indicated that the hydrophilicity and wettability of SCMC-5 3D-printed ODFs were greater than the others. The significant decrease in water contact angle of SCMC-5 3D-printed ODFs may be related to the film surface roughness. This result was in agreement with the AFM studies which showed the highest surface roughness of SCMC-5 3D-printed ODFs. According to Wenzel's model, the equation predicts that if the hydrophilic surface becomes roughened, the surface energy of the wetting process will be increased. Then, the rough surfaces are more easily wetted and it becomes more hydrophilic. Thus, the surface roughness may decrease the contact angle for a droplet on a hydrophilic surface [45,46]. The significant decrease in water contact angle of SCMC-5 3D-printed ODFs is also related to the film disintegration. However, the 3D-printed ODFs still did not disintegrate or turn into a gel layer after 30 s of the water contact angle measurements. This may be due to the difference between the temperature and solvent volumes in the contact angles experiment and those in the disintegration experiment. For the contact angles experiment, a small volume (only 20 µL) of water was used as solvent. Thus, the experiment showed that the film would be no longer a film after 1.5–2 min; whilst the temperature in the disintegration experiment was controlled at 37 °C to represent the temperature in the oral cavity, which may facilitate the disintegration process as well.

## 4. Conclusions

Our study has investigated the feasibility of using five different kinds of hydrophilic polymers as printing materials for the fabrication of 3D-printed ODFs and characterized the physicochemical and mechanical properties of the 3D-printed ODFs. The results demonstrate that all the HPMC E15, HPMC E50, HMP, SCMC, and HEC printing gels had non-Newtonian pseudoplastic behaviors which allow the extrusion and printing processes. To conclude, 20% *w*/*v* of HPMC E15, 15% *w*/*v* of HPMC E50, 15% *w*/*v* of HMP, and 5% *w*/*v* of SCMC exhibited good printability and shape fidelity with satisfying dimensional accuracy and were the most suitable concentrations used for extrusion printing of the various 3D structures pharmaceutical dosage forms through our custom-built in-house syringe extrusion 3D printer. Furthermore, another finding of this study was that the 3D-printed ODFs with 5% *w*/*v* of SCMC (SCMC-5) showed the best ODFs properties with significantly the most rapid disintegration time of 2.08 s. The presence of porous structure and roughness surface the SCMC-5 3D-printed ODFs may be beneficial for the penetration of the water or simulated salivary fluid into the 3D-printed ODFs, thus enhancing the disintegration rate of films. Consequently, this study suggests that SCMC is an ideal candidate for fabrication of the ODFs by using syringe extrusion 3D printing technology and it also could be used as a 3D printing material for the other dosage forms. However, for the application in practical use, these SCMC-5 3D-printed ODFs still need further development, especially in terms of their mechanical properties. The use of plasticizers (e.g., glycerin, propylene glycol, polyethylene glycol, etc.) needs to be considered to be incorporated into the printing gels in order to enhance the handling safety of the films. In addition, upon the incorporation of the drug in further study, the viscosity properties and particle sizes of the drug-loaded printing gels as well as some of the printing parameters need to be taken into account and optimized in order to ensure good extrudability and printability. The majority of drug compounds can chemically interact with polymer molecules, thus affecting almost all of the investigation tests that have been performed in this study.

## Figures and Tables

**Table 1:** Compositions of each printing gel.

Formulation Code	Polymer (% *w*/*v*)					Methylene Blue (*%* *w*/*v*)
	HPMC E15	HPMC E50	HMP	SCMC	HEC	
E15-15	15.0	-	-	-	-	1.0
E15-17.5	17.5	-	-	-	-	1.0
E15-20	20.0	-	-	-	-	1.0
E50-12.5	-	12.5	-	-	-	1.0
E50-15	-	15.0	-	-	-	1.0
E50-17.5	-	17.5	-	-	-	1.0
HMP-12.5	-	-	12.5	-	-	1.0
HMP-15	-	-	15.0	-	-	1.0
HMP-17.5	-	-	17.5	-	-	1.0
SCMC-3	-	-	-	3	-	1.0
SCMC-4	-	-	-	4	-	1.0
SCMC-5	-	-	-	5	-	1.0
HEC-2	-	-	-	-	2	1.0
HEC-3	-	-	-	-	3	1.0
HEC-4	-	-	-	-	4	1.0

**Table 2:** Rheological and printability parameters of different concentrations of HPMC E15, HPMC E50, HMP, SCMC, and HEC formulations.

Formulation Code	Viscosity (Pa·s)	Flow Behavior Index	Consistency Coefficient $\left(Pa\cdot s^{n}\right)$	Shape Fidelity Factor	Diameter of Printed Filament (mm)
E15-15	8.20 ± 0.07	0.90	12.06	1.12 ± 0.03	1.32 ± 0.04
E15-17.5	15.42 ± 0.19	0.89	23.80	1.09 ± 0.02	1.03 ± 0.07
E15-20	26.27 ± 0.46	0.81	54.56	1.06 ± 0.01	0.57 ± 0.07
E50-12.5	15.11 ± 0.26	0.57	72.16	1.11 ± 0.02	0.85 ± 0.12
E50-15	26.03 ± 0.64	0.44	187.54	1.06 ± 0.03	0.53 ± 0.03
E50-17.5	36.48 ± 0.72	0.31	402.90	NA	NA
HMP-12.5	16.60 ± 0.69	0.49	100.95	1.06 ± 0.02	1.18 ± 0.09
HMP-15	34.26 ± 0.94	0.34	341.66	1.06 ± 0.02	0.59 ± 0.01
HMP-17.5	43.68 ± 1.13	0.30	494.08	1.03 ± 0.01	0.41 ± 0.02
SCMC-3	9.26 ± 0.06	0.27	114.58	1.02 ± 0.02	1.25 ± 0.13
SCMC-4	21.74 ± 1.19	0.19	341.90	1.08 ± 0.02	0.88 ± 0.08
SCMC-5	39.44 ± 3.03	0.13	723.44	1.03 ± 0.01	0.56 ± 0.02
HEC-2	10.25 ± 0.11	0.23	141.81	1.03 ± 0.02	1.03 ± 0.06
HEC-3	24.69 ± 0.71	0.16	413.24	1.07 ± 0.02	0.77 ± 0.09
HEC-4	44.46 ± 0.62	0.07	963.39	1.04 ± 0.01	0.56 ± 0.05

Note: NA (Not applicable) means the printing formulations could not extrude through the nozzle.

**Table 3:** Atomic force microscopy parameters of 3D-printed ODFs.

AFM Parameters	Formulation			
	E15-20	E50-15	HMP-15	SCMC-5
Average surface roughness (Sa, nm)	1.51	2.12	6.83	8.83
RMS roughness (Sq, nm)	1.13	1.61	5.34	6.96
Maximum peak height (Sp, nm)	11.50	16.94	27.61	30.09
Maximum pit depth (Sv, nm)	6.33	12.02	30.39	32.28

**Table 4:** Weight and thickness of 3D-printed ODFs.

Formulation	Thickness (µm ± SD)	Weight (mg ± SD)
E15-20	88.93 ± 3.60 ^a^	40.46 ± 2.04 ^a^
E50-15	63.87 ± 4.95 ^b^	29.64 ± 1.91 ^b^
HMP-15	46.80 ± 5.47 ^c^	29.88 ± 2.61 ^b^
SCMC-5	18.73 ± 2.25 ^d^	9.98 ± 0.54 ^c^

For each test, average values with the same letter are not significantly different. Thus, average values with the different letter, e.g., ‘a’ or ‘b’ or ‘c’ or ‘d’, are statistically different (*p* < 0.05).

**Table 5:** Mechanical characteristics of 3D-printed ODFs.

Formulation	Normalized Tensile Strength (MPa)	Normalized Elongation (%)	Young’s Modulus (MPa)
E15-20	2.52 ± 0.07 ^a^	2.10 ± 0.22 ^a^	245.63 ± 17.36 ^a^
E50-15	2.96 ± 0.40 ^a^	2.91 ± 0.21 ^b^	279.99 ± 19.92 ^b^
HMP-15	2.32 ± 0.32 ^a^	2.23 ± 0.23 ^a^	310.74 ± 14.36 ^c^
SCMC-5	2.37 ± 0.37 ^a^	3.76 ± 0.38 ^c^	333.37 ± 4.16 ^c^

For each test, average values with the same letter are not significantly different. Thus, average values with the different letter, e.g., ‘a’ or ‘b’ or ‘c’ are statistically different (*p* < 0.05).

**Table 6:** In vitro disintegration time and water contact angles of 3D-printed ODFs.

Formulation	Disintegration Time (s)	Normalized Disintegration Time (s)	Water Contact Angle (°)	
			Initial	After 30 s
E15-20	49.85 ± 14.28 ^a^	10.62 ± 2.83 ^a^	62.4 ± 6.2	61.8 ± 5.2
E50-15	24.08 ± 5.88 ^b^	7.11 ± 1.25 ^b^	52.5 ± 0.2	52.4 ± 1.7
HMP-15	6.48 ± 1.07 ^c^	2.63 ± 0.38 ^c^	58.4 ± 1.9	56.9 ± 1.3
SCMC-5	2.02 ± 0.14 ^c^	2.08 ± 0.28 ^c^	51.5 ± 2.6	45.1 ± 2.6 *

For disintegration test, average values with the same letter are not significantly different. Thus, average values with the different letter, e.g., ‘a’ or ‘b’ or ‘c’, are statistically different (*p* < 0.05). For the water contact angle test, an asterisk symbol (*) indicates significant difference between water contact angle at initial and after 30 s (*p* < 0.05) in each formulation.

## Supplementary Materials

The following are available online at https://www.mdpi.com/article/10.3390/polym13203454/s1, Figure S1: Printing filaments of (a) E15-20, (b) E50-15, (c) HMP-15, (d) SCMC-5, and (e) HEC-4.
