# Supplementary material for: Characterization of Hydrophilic Polymers as a Syringe Extrusion 3D Printing Material for Orodispersible Film
Source: Polymers (Basel). 2021 Oct 9;13(20):3454. doi: 10.3390/polym13203454 (PMC8540066; doi:10.3390/polym13203454)
Supplement: Supplementary file 1 [file polymers-13-03454-s001.zip › polymers-1397196-supplementary.pdf]

polymers

MDPI

Supplementary Materials: Characterization of Hydrophilic Polymers as a Syringe Extrusion 3D Printing Material for Orodispersible Film

Pattaraporn Panraksa [1], Sheng Qi [2], Suruk Udomsom [3], Pratchaya Tipduangta [1], Pornchai Rachtanapun [4,5], Kittisak Jantanasakulwong [4,5] and Pensak Jantrawut [1,5,*]

1 Department of Pharmaceutical Sciences, Faculty of Pharmacy, Chiang Mai University, Chiang Mai 50200, Thailand; pattaraporn.prs@gmail.com (P.P.); ptipduangta@gmail.com (P.T.)
2 School of Pharmacy, University of East Anglia, Norwich, Norfolk NR4 7TJ, UK; sheng.qi@uea.ac.uk
3 Biomedical Engineering Institute, Chiang Mai University, Chiang Mai 50200, Thailand; suruk_u@cmu.ac.th
4 Division of Packaging Technology, School of Agro-Industry, Faculty of Agro-Industry, Chiang Mai University, Chiang Mai 50100, Thailand; pornchai.r@cmu.ac.th (P.R.); jantanasakulwong.k@gmail.com (K.J.)
5 Cluster of Agro Bio-Circular-Green Industry (Agro BCG), Chiang Mai University, Chiang Mai 50100, Thailand
* Correspondence: pensak.amuamu@gmail.com or pensak.j@cmu.ac.th; Tel.: +6653944309

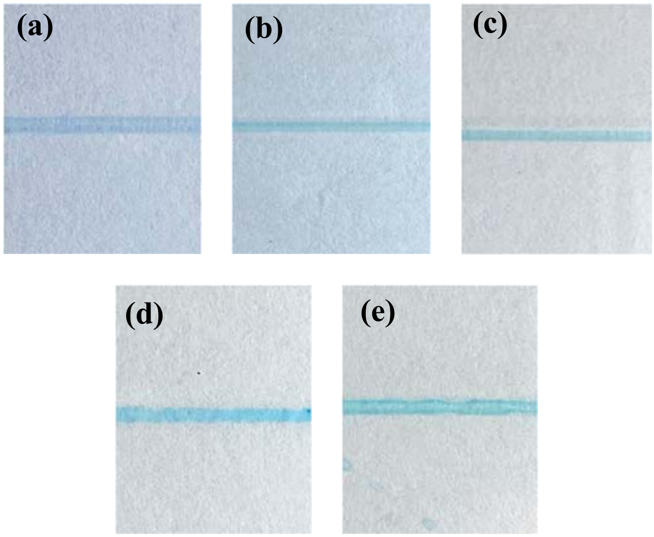

Figure S1. Printing filaments of (a) E15-20, (b) E50-15, (c) HMP-15, (d) SCMC-5, and (e) HEC-4.
